# Lifetime Maximization via Hole Alleviation in IoT Enabling Heterogeneous Wireless Sensor Networks

**DOI:** 10.3390/s17071677

**Published:** 2017-07-21

**Authors:** Zahid Wadud, Nadeem Javaid, Muhammad Awais Khan, Nabil Alrajeh, Mohamad Souheil Alabed, Nadra Guizani

**Affiliations:** 1 University of Engineering & Technology, Peshawar 25000, Pakistan; zahidmufti@nwfpuet.edu.pk; 2 Capital University of Science and Technology, Islamabad 44000, Pakistan; 3 COMSATS Institute of Information Technology, Islamabad 44000, Pakistan; awaixmuhammad051@gmail.com; 4 Biomedical Technology, Department College of Applied Medical Sciences, King Saud University, Riyadh 11633, Saudi Arabia; nabil@ksu.edu.sa (N.A.); salabed@ksu.edu.sa (M.S.A.); 5 Department of Electrical and Computer Engineering, Purdue University, West Lafayette, IN 47907, USA; nguizani@purdue.edu

**Keywords:** Internet of Things Wireless Sensor Networks (IoTWSNs), traffic load, energy consumption, energy hole, network lifetime, End2End Delay (E2ED), routing, linear programming

## Abstract

In Internet of Things (IoT) enabled Wireless Sensor Networks (WSNs), there are two major factors which degrade the performance of the network. One is the void hole which occurs in a particular region due to unavailability of forwarder nodes. The other is the presence of energy hole which occurs due to imbalanced data traffic load on intermediate nodes. Therefore, an optimum transmission strategy is required to maximize the network lifespan via hole alleviation. In this regard, we propose a heterogeneous network solution that is capable to balance energy dissipation among network nodes. In addition, the divide and conquer approach is exploited to evenly distribute number of transmissions over various network areas. An efficient forwarder node selection is performed to alleviate coverage and energy holes. Linear optimization is performed to validate the effectiveness of our proposed work in term of energy minimization. Furthermore, simulations are conducted to show that our claims are well grounded. Results show the superiority of our work as compared to the baseline scheme in terms of energy consumption and network lifetime.

## 1. Introduction

The emergence of Internet has increased enormously the connectivity of human beings at unprecedented scale. However, the rapid growth of short range networks including; Wireless Sensor Networks (WSNs) Bluetooth, Radio Frequency Identification (RFID), Wireless Fidelity (WiFi), ZigBee, etc.; the interconnection between numerous devices is inevitable [1]. Now, it is obvious that devices will be interconnected and sense, share, gather information via sequence of communication in multi-hop manner without the involvement of humans. The devices could be distinct in terms of capabilities such as processing, intelligence, e.g., sensors, smart phones, etc. The interconnection of devices along with humans has led to the advent of new paradigm known as Internet of Things (IoT). IoT embeds intelligence in our environment by transforming gathered data into intelligent information. WSN has been considered as an essential component of IoT. However, this crucial part has few constraints such as energy of the sensing device that must be utilized efficiently for effective operations in the network [2]. These sensing devices have the abilities to sense, monitor, process signals and allow wireless communication [3]. Due to the unique features of sensors nodes; various applications are now possible in accessible, non-accessible and remote areas. Applications include military surveillance, temperature sensors, traffic monitoring and environments, etc. [4]. WSNs composed of small size sensor nodes which are randomly or deterministically deployed in the desired sensing area. The purpose of the deployment of sensing devices is to gather information of interest and transmit it to the base station. However, one of the major factors need to be considered that is battery of the sensor node. The lifetime of the network totally depends upon the distribution of the data traffic load on the network nodes [5].

In this regard, an energy efficient routing strategy is desired in order to optimize the use of node battery which will directly enhance the network operation time. Without an optimal routing mechanism, uneven energy dissipation of network nodes degrades the network performance. The imbalanced transmissions lead to quick energy depletion of intermediate nodes resulting in pre-mature end of network. This immature end of network lifetime is because of energy dissipation of intermediate nodes [5]. However, uneven energy consumption of intermediate nodes is not the only reason for hole occurrence. The reasons for holes occurrence includes but not only are: non uniform or random nodes deployment, sensor nodes lack physical capabilities and turn into sleep mode in order to save energy, mobility of nodes which breaks the linkage between nodes and excess amount of neighbor requests.

WSNs can be classified into homogenous and heterogeneous networks. In former, all sensor nodes have same capabilities such as same energy, processing and sensing range. While in later, network nodes capabilities varies in terms of energy, processing, etc. [6]. Whether network is homogeneous or heterogeneous, before designing a routing protocol following factors must be taken into consideration for achieving optimal results.

- Routing protocol must has a design that is able to find the least energy consuming path.
- Controlling the traffic is major contribution in any routing mechanism. Due to high traffic, nodes consume excess amount of energy.

In order to cater aforementioned challenges, research community devoted its efforts to maximize the network lifetime. In this regard, routing strategies are classified into flat network routing, location based routing and hierarchical based routing. In flat network routing sensor nodes work together in order to perform homogeneous routing tasks [7]. In location based routing, path is computed through the location information of the sensor nodes [8]. In hierarchical based routing, the network is divided into groups such that to minimize the energy and improves the scalability. Hierarchical based routing can be cluster or ring routing. Ring routing is the most scalable routing technique in WSNs [9]. Ring routing uses greedy geographic mechanism for scalable and time efficient routing [10]. Following are the advantages of ring routing but not the only:

- Ring routing heavily relies on minimal broadcasting to ensure fast data delivery.
- Applicable for event driven applications.
- Does not require information about the motion of the sink.

Many routing mechanisms have been proposed in WSNs for maximizing the network performance by minimizing the energy consumption of the sensor nodes. To alleviate hole for maximizing lifetime of the network, Jan et al. [11] introduce Balance Energy Consuming and Hole Alleviating (BECHA) and Energy Aware Balance Energy Consuming and Hole Alleviating (EA-BECHA) algorithms to balance load distribution among nodes as well as efficient energy allocation to the nodes in order to alleviate hole. An analytical model is proposed by Ren et al. [12] for maximizing the network lifetime via energy hole alleviation. The proposed work divides the network into small regions. With the death of nodes in the region close to the sink, nodes lie above the death region act as data forwarder for transmission of data to the sink. Both proposed algorithms able to alleviate energy hole around the sink however unable to tackle void hole. Moreover, high traffic load on nodes in the lower region increases the energy consumption of the nodes. Thus there is a need of routing mechanism that is able to control the traffic load among the nodes as well as to recover the nodes from void region.

In this paper, we proposed a routing technique [13] for WSNs that consists of Heterogenous nodes. An optimal data path is computed in order to utilize efficiently scarce resource like energy for prolongation of network lifespan. Also the division of network is performed to deploy heterogeneous nodes to recover from void hole. The details of our routing strategy are given in Section 4.

**Contributions**: The contributions of our work in this paper are: (1) the use of the divide and conquer algorithm for obtaining optimal number of sectors in the network; (2) For network lifetime prolongation, minimal energy dissipation route is obtained; (3) In order to ensure that scarce resource (energy) is utilized effectively, data load is estimated to avoid overburden of data at intermediate nodes; (4) An optimal bandwidth is allocated for throughput maximization which is validated via linear programming using graphical analysis. The following enlisted steps are followed in our proposed work.

- Network division into optimal number of sectors
- Minimum energy path computation
- Traffic load estimation
- Energy minimization through feasible region
- In order to avoid congestion at super node, optimal allocation of bandwidth
- Simulations to prove the legitimacy of our work.

The rest of the paper is organized as follows: in Section 2, we discuss some of the previous techniques related to WSNs. Proposed strategy is discussed in Section 3. In Section 4, simulations are performed. Finally, conclusion and future work are written in Section 5.

## 2. Related Work

A lot of work has been done in maximizing the performance of the network via proposing different routing techniques. Sahoo et al. introduce a distributed coverage hole repair algorithm (HORA) [14]. The proposed algorithm considers the movement of sensor nodes. Each node checks it status whether it is Cross Triangles (CT), Hidden Cross Triangles (HCT) or Non Cross Triangle (NCT) in order to minimize overlapping region. Neighbor node with highest overlapping region will move towards coverage hole to fill the gap. HORA helps to improve network lifetime and maximizes the throughput but its application in delay sensitive area degraded due to hole repairing process.

Energy hole is one of the major factor which minimizes the network lifetime. Death of the nodes in the innermost region of the sink minimizes the network performance. A well known “Load Balancing Technique” (LBT) [15] aims to recover energy hole by adjusting the transmission power of sensor nodes. The proposed technique unable to highlight energy hole problem. Khan et al. [16] alleviate energy hole through deployment of super nodes in the region close to the sink. The nodes near the sink turn on their scheduling power when all the data are gathered by super nodes on the boundary of the sink region. The scheme able to alleviate energy hole however the problem of void hole still exists which decreases the performance of the network. In order to control uneven energy consumption Li et al. [17] propose a ring model and define the per node traffic load and energy consumption. According to the observation, sensor nodes near the sink relay more traffic as compared to sensor nodes far from the sink. Based on their analysis sensor nodes in the region close to the sink has high energy consumption thus die out early which creates energy hole around the sink. A cluster based model “Energy-efficient HOle Removing Mechanism” (E-HORM) is presented by Rasheed et al. [18] to overcome energy hole. In the propose scheme sleep scheduling mode is adopted for energy saving. Maximum distance nodes are selected for calculating the maximum energy for transmission of data. An energy threshold “$E_{th}$” is defined. If a node energy level falls below this $E_{th}$, it cannot transmit data. The propose scheme able to maximize network lifetime and stability period in expense of delay. Jewel et al. [19] propose an “Improved Hole Detection Healing and Replacing Algorithm for optimal coverage in Wireless Sensor Networks” (IHDHRA) in order to maximize network lifetime via increasing the probability of packet loss. The propose algorithm uses nodes replacement strategy in order to avoid hole. High energy level node is selected for replacement such that the overall coverage is not disturbed. Maximum lifetime is achieved. However scalability issue and robustness decreases the performance.

For energy balancing, an “Energy Balance Mechanism” (EBM) is proposed by Ekal et al. [20] to maximize the performance of the network. A corona based model is considered such that to balance the energy among the nodes. Required energy of every node is calculated based on its initial energy in respective corona and corona load. The proposed model aim to improve network lifetime. However extra energy is provided to these nodes which is not a best solution for lifetime maximization. Lu et al. [21] propose an “ Energy-Efficient Data Sensing and Routing in unreliable energy-harvesting wireless Sensor network” (EEDSRS) that performs both data sensing and data routing. EEDRS is performed in three steps. In first step an adaptive exponential weighted moving algorithm is proposed for estimation of link quality. Second, a distributed energy efficient rate allocation is performed for data sensing and routing for lifetime maximization through optimal data sensing rate. In last, data is routed through the links via energy efficient path. The algorithm aims to optimize the network performance but it is purely a MAC layer protocol which increases the complexity. A mixed transmission strategy is propose in [22] for energy balancing. The scheme considers not only the distance and energy of nodes but also considers the link reliability and number of neighbor nodes in selecting the relay nodes. The scheme performs better in terms of maximizing the network lifetime in expense of delay. Kumar et al. [23] propose a position based routing algorithm for lifetime maximization. The algorithm uses the Forwarding Search Space (FSS) to control retransmission. The function for the selection of forwarder node is based upon its degree, distance and angle. Each time, different set of sensor nodes are selected from this function for data transmission for energy balancing. Due to greater number of calculations involve in selection of data forwarder it delays the performance of the network.

Data aggregation is one of the useful concept in WSNs. Many researchers try to minimize unnecessary transmission in order to minimize energy consumption. For this reason an “Energy Efficient Ant Colony algorithm” (EEAC) is proposed by Lin et al. [24] such that to gather the data from sensor nodes. In the propose scheme sensor nodes use the remaining energy for finding the next hop data forwarder. The scheme helps to minimize energy consumption and increases the network lifetime in expense of robustness and scalability issue. Liu [25] present a transmission strategy called “An Optimal-Distance based Transmission Strategy for lifetime maximization of wireless sensor networks” (ODTS) using ant colony optimization technique. In ODTS, every ant moves from one corona to other corona in order to transmit data to the sink. For lifetime maximization an optimal path solution is developed for energy minimization through Most Energy Efficient Distance (MEED) and energy balancing through Most Energy Balanced Distance (MEBD). The propose scheme prolongs the network lifetime and minimizes the energy consumption in sparse area network however in dense area network its performance is effected due to high traffic load on ants which increases the energy consumption of the network and tends to decrease network lifetime.

Energy consumption is one of the major factor which decreases the performance of the network. An energy efficient routing protocol is must in this case. For energy efficiency Ghaffari et al. [26] introduce a routing mechanism that selects minimum hop counts for the selection of forwarder nodes. It selects nodes that have minimum distance from the sink. Moreover it also identifies the link quality while selecting minimum hop counts. Network lifetime is improved in dense area network however its performance is degraded in sparse area network due to the unavailability of forwarder nodes. In order to maximize network performance Jin et al. [23] propose an “Energy Efficient tree based Data Collection Protocol” (EEDCP-TB) for data gathering using cascading time mechanism by efficiently allocating time slots in order to save nodal energy. EEDCP-TB helps in maximizing network lifetime in expense of delay. “Lifetime Maximizing Dynamic Energy efficient routing protocol” (LMDE) is presented by Bhattachargee et al. [27] to optimize network performance. The routing mechanism uses the remaining energy of nodes for data forwarding. The scheme improves the network lifetime however fails to control the scalability and data redundancy.

In existing state of the art [12,25,28,29], the authors have adjusted transmission ranges for balancing energy depletion across the network nodes. However, we have adopted the divide and conquer strategy for estimation data load to adjust the transmission power accordingly. To avoid the congestion over the intermediate nodes, an optimal bandwidth is allocated to super nodes according to the data load for improving throughput. Moreover, we have incorporated the computation of data at each hop for avoiding overload of traffic at intermediate nodes. The description of our proposed algorithm is presented in Section 3. Table 1 shows routing techniques comparison.

### 2.1. An Overview of Baseline Schemes

For simplicity, we have provided an overview of our baseline schemes in this section. In [25], the authors exploited ACO for balancing energy consumption among the network nodes. The network is divided into number of regions where ants are deployed to gather data from the desired network field. This protocol presents two functions named Most Energy Efficient Distance (MEED) for ensuring effective energy depletion for maximizing the operational time of the network. ACO is used to find out an optimal distance via pheromone intensity and heuristic desirability that consists of data load and transmission range. The proposed strategy ensures balanced energy consumption resulting in optimal network lifespan. [12] exploits the ring routing through dividing the network into small sectors for estimating data load at each node. Transmission ranges are adjusted in order to avoid the cyclic selection of the forwarder node. Due to the converge cast nature of the algorithm, data load increases viua moving towards the destination resulting in immature end of network. This mechanism adjusts transmission range when nodes in lower coronas’ are dead. Due to this factor, as network progresses, transmissions over long distances deplete energy of the node very quickly resulting in undesired end of the network with plenty of nodes alive in the network.

A new stable election based routing algorithm (N-SEP) proposed to preserve energy of the network [28]. Authors have exploited heterogeneity in cluster based network for saving energy over long distances. Cluster heads are randomly elected through weighted probability in which every node above certain threshold is illegible to become a cluster head. The probability for the nomination of head node is computed as follows:

(1) $$Th_{norm}\left(r\right)=\left\{\begin{array}{cc} \frac{P_{N}\left(r\right)}{\left(1-P_{N}\left(r\right)\right)\left(r\;mod\;\frac{1}{P_{N}\left(r\right)}\right)}, & \mathrm{if}\;N\in N_{s}\\ 0, & \mathrm{otherwise} \end{array}\right.$$

(2) $$Th_{norm}\left(r\right)=\left\{\begin{array}{cc} \frac{P_{A}\left(r\right)}{\left(1-P_{A}\left(r\right)\right)\left(r\;mod\;\frac{1}{P_{A}\left(r\right)}\right)}, & \mathrm{if}\;N\in A_{s}\\ 0, & \mathrm{otherwise} \end{array}\right.$$

where $N_{s}$ and $A_{s}$ are the sets of normal nodes and forwarder nodes respectively.

After the selection of a node as a cluster head, the member nodes are decided based on the minimum distance with respect to the destination. This technique achieves considerable amount of energy efficiency. An advanced version of N-SEP is proposed named a prolong stable election routing algorithm (P-SEP) [29] for limited heterogeneous WSNs. Same like N-SEP, heterogeneous network is considered where normal nodes and advance nodes are considered in the network having different energy levels. The energy level of advance nodes is greater than the normal nodes. The selection of cluster head is based upon the weighted probability using the following equations:

(3) $$p_{norm}=\frac{p}{1+\alpha m}$$

(4) $$p_{adv}=\frac{p\left(1+\alpha m\right)\bar{E_{n}\left(r\right)}}{\left(1+\alpha m\right)\bar{E_{adv}}}$$

where $p_{norm}$ and $p_{adv}$ are the weighted probabilities for normal and advance nodes, respectively. $\varepsilon_{n}\left(r\right)$ is the average energy of nodes including normal nodes and advance nodes in $r_{th}$ round. Moreover the selection of cluster head is based upon the threshold value. The threshold value for the selection of cluster head is given as:

(5) $$Th_{norm}=\left\{\begin{array}{cc} \frac{p_{norm}}{\left(1-P_{norm}\right)\left(r\;mod\;\frac{1}{p_{norm}}\right)}E_{N_{i}}\left(r\right), & \mathrm{if}\;N\in N_{s}\\ 0, & \mathrm{otherwise} \end{array}\right.$$

(6) $$Th_{adv}=\left\{\begin{array}{cc} \frac{p_{adv}}{\left(1-P_{adv}\right)\left(r\;mod\;\frac{1}{p_{adv}}\right)}\frac{1}{E_{N_{i}}\left(r\right)}, & \mathrm{if}\;N\in A_{s}\\ 0, & \mathrm{otherwise} \end{array}\right.$$

$N_{s}$ and $A_{s}$ are the set of normal nodes and advance nodes that are declined to become the cluster heads lasts $\frac{1}{p_{norm}}$ and $\frac{1}{p_{adv}}$, respectively. An optimal and having less threshold value; node is nominated cluster head for delivering the data towards forwarder node. Moreover, an optimal path is selected for sending data towards forwarder node with less energy consumption. An optimal path is chosen via calculating the distance of head node from forwarder and vice versa. Nodes choose nearest head node while head node elects nearest forwarder for data forwarding. This algorithm achieved energy efficiency and network lifetime.

### 2.2. Problem Statement

The balancing of load in order to minimize the energy consumption is one of most researched topics in WSNs. In this regard, various routing mechanisms for network layer have been proposed [12,25,28,29]. However, the proposed strategies are not as energy efficient as desired. First of all the adjustment of transmission range is not helpful in distributing the nodal traffic load in converge-cast routing algorithms. The data traffic from outermost coronas is sum up and passed on to lower corona until it reaches the sink. With the decrement of each corona, number of data packets increases which means more energy will be consumed in transmission and reception. Ultimately, node placed closer to sink quickly depletes its energy and energy hole occurs. Secondly, the load is balanced among the network nodes when the node density ρ is high, but in sparse deployment, the occurrence of void hole is inevitable. Thirdly, the authors have not adopted any sort of mechanism to ensure that during the transmission range adjustment, multiple number of nodes will not chose the same forwarder node. Due to the ignorance, energy holes occur resulting in immature end of the network lifetime while a network have ample of sensing devices alive away from the sink. In order to cater the aforementioned problems, we have proposed a new routing protocol. The details are as follows:

## 3. LiMHA

Energy is one of the scarce resources in WSNs; therefore, it needs to be utilized efficiently for effective network operations. The uneven distribution of data load on intermediate nodes is among the leading factors which ends the battery of a node immaturely. In this regard, we propose a routing approach Lifetime Maximization via Hole Alleviation (LiMHA) in which heterogeneity of network is exploited to avoid the quick depletion of nodes energy. The details of the proposed work are as follows:

### 3.1. Network Model

**Figure 1:**
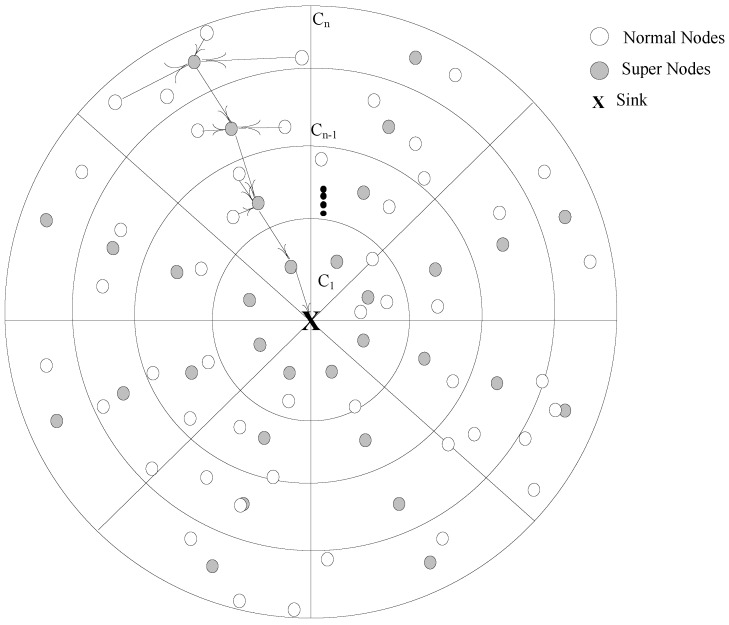
Proposed Network Model.

In order to show relationship between various components of the network; we have made numerous assumptions such as; for monitoring, sensing and gathering information from the desired network field, *N* number of heterogeneous nodes are randomly deployed having fixed energy and irreplaceable battery [12]. There are two kinds of nodes: normal nodes (NN) and super nodes (SN); NN are supposed as low energy nodes while SN are powered with high energy battery. Hence, the total number nodes are deployed in the network; $\sum_{i=1}^{N}NN+SN$. Nodes remains static and have various transmission ranges that could be r≤R. The network field is assumed having radius *R* with thickness ξ; which is further divided in to $C_{n}$ of concentric coronas denoted as set like $\left\{C_{1},C_{2},....,C_{n}\right\}$. In order to exploit the features of divide and conquer, $C_{n}$ are decomposed in to number of small sectors *S* where each *S* has node density ρ including a SN. Sink is placed at the center of the network field and data is passed on to sink in a converge-cast manner. The quality of links purely depends upon the degree of closeness to the sink, more the node close is, higher will be the link quality. The network model is illustrated in Figure 1.

### 3.2. Network Configuration

For communication, a reliable and well stable infrastructure is always desired in a network. By keeping in mind the fact that sensor nodes are freshly planted, an infrastructure is required to share and communicate information in the network. The configuration phase starts at the beginning of each round, where every node acquire information about its sector *S* that SN is in its vicinity to transmit data or it has to deliver data via a neighbor node known as NN. After gaining the information, NN establishes a data link with the respective node according to the information. This process repeats until all nodes acquire the desired information.

### 3.3. Energy Model

To portray the dissipation of energy of the network nodes; a widely accepted radio model for WSNs is adopted in our proposed work [12]. A node consumes energy while transmitting *k* number of bits over distance *d* is depicted as shown in Equation (7).

(7) $$E_{tx}\left(k,d\right)=\left\{\begin{array}{cc} kE_{elec}+k\epsilon_{fs}d^{2}, & \mathrm{if}\;d\leq d_{o}\\ kE_{elec}+k\epsilon_{amp}d^{4}, & \mathrm{otherwise} \end{array}\right.$$

where $E_{tx}$ is the required transmission energy for effectively delivering data at the destination, $E_{elec}$ illustrates the battery utilization in circuitry operations like analog or digital coding, modulation and spreading of a signal in free space (in communication range) $\epsilon_{fs}$ over $d^{2}$ and multi-path environment where the probability of fading, scattering and attenuation is high as compared to $\epsilon_{fs}$ over $d^{4}$.

(8) $$E_{rcv}=kE_{elec}$$

where $E_{rcv}$ depicts the energy dissipated while receiving *k* bits.

### 3.4. Traffic Load Estimation

Data traffic management is a key factor in optimizing the network lifetime. To manage the data traffic, the network is divided in to small regions known as sectors *S*. Due to the division of network field, now it is easy to estimate the data load at each *S* by computing the node ρ. As it is well known that downstream nodes bear high data load while upstream nodes just forward data of their own *S*. Now, it can be easily estimated that how much data packets are generated from the current *S*. Let assume $S_{n}$ be a sector close the sink having distance *d* from the sink. The total traffic generated by sector $S_{n}$ is the sum of traffic generated by previous sector $S_{n+1}$. The mathematical formulation given as follows [12]:

(9) $$TF_{j_{S_{n}}}=N_{S_{n}}+N_{S_{n-1}}+N_{S_{n-2}}+.....+N_{S_{1}}$$

If, the average traffic load on $S_{n}$ is desired to compute that Equation (9) can be written as:

(10) $$ATF_{S_{n}}=\frac{TF_{j_{S_{n}}}}{N_{S_{n}}}$$

**Figure 2:**
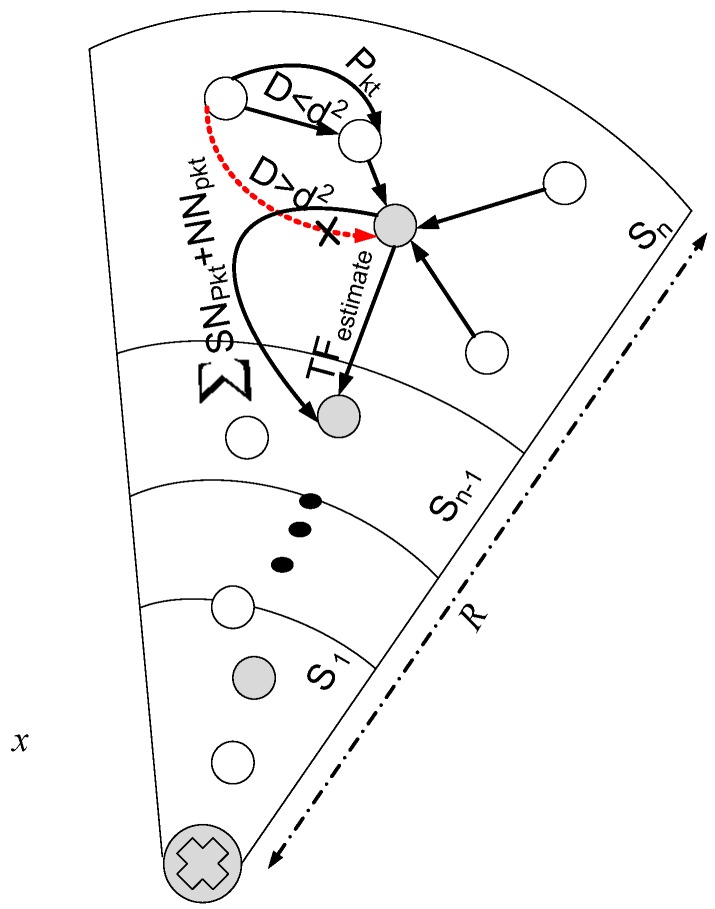
Traffic Load Estimation and Data Transmission.

ATF is the average traffic load on a sector close to the sink, thus, we can use ATF for finding the traffic load on each sector [12].

(11) $$ATF_{jS_{n}}=\left\{\begin{array}{cc} \left(T_{1}+1\right)+\frac{T_{1}\left(1+T_{1}\right)r}{2x}, & \mathrm{if}\;x\geq \xi \\ \frac{1}{2}\left(T_{2}+2\right)\xi^{2}\theta \rho +\frac{1}{2}T_{2}r\xi \theta \rho \left(T_{2}+1\right), & \mathrm{otherwise} \end{array}\right.$$

where $T_{1}=\frac{\left(R-d\right)}{r}$ and $T_{2}=\frac{\left(R-\xi \right)}{r}$ [12]. The traffic load working mechanism is discussed in Algorithm 1. In which it is shown that before the initialization of each round as discussed in Section 3.2 every node acquires its distance from the neighbor node in order to adjust its transmission range. By using Equation (11) load is estimated and then accordingly SN is selected with in the vicinity of the adjusted transmission range for establishing link with good quality as shown in Figure 2. At each hop, it is calculated that every node measures its data load, if the data is very high that NN will forwards data to SN directly else via neighbor nodes. A point here must be noted that nodes within the communication range of sink will transmit the generated data packet directly without the help of any intermediate  node.

**Algorithm 1:** Calculating Traffic Load and Energy Consumption at each round **Input**: Network range *R*, transmission range *r* between sensor, NN normal node, SN super node, node density ρ etc. **Output**: for a node *i*∈ {NN} determine the traffic load $tl_{i}{}^{r_{o}}$, energy consumption $e_{i}{}^{r_{o}}$ at round $r_{o}$ **1**: Initialize Parameters **2**: for round $r_{o}$ **3**: for each node *i*∈ {NN} and at each round $r_{o}$ calculate distance $d_{\left(i,j\right)}$ where node *i* is node of sector *S* and node *j* is node of sector $S_{n}$ also *n* is number of sectors **4**: if $d_{\left(i,j\right)}$ ≤ *r* **5**: send data of node *i* to node *j*. Calculate the traffic load from Equation (11) and energy consumption for data transmitting and receiving from Equations (7) and (8), respectively. **6**: else **7**: Find SN in each sector **8**: if $d_{\left(NN,SN\right)}$ ≤ *r* **9**: send data of NN to SN **10**: calculate the traffic load and energy consumption for data receiving for SN and data transmitting for NN **11**: else **12**: SN receive the data by itself **13**: calculate the traffic load and energy consumption for data receiving for SN **14**: while sink receive data **$d_{o}$** **15**: Calculate the overall energy consumption, lifetime of nodes at round $r_{o}$

### 3.5. Description of Algorithm 1

For better understanding, Algorithm 1 has been elaborated briefly step wise in this section. Input shows that control parameters need to be defined before the initialization of an algorithm. Transmission range and network radius along with NN and SN deployment. After the initialization of network parameters in step 1, which is deployment of nodes, sink and network configuration then step 2 is initiated known as loop of rounds. At step 3, each node computes its distance from every other node in order to find out minimal route to forward its data packets towards the destination. Also NN calculate distance from NN and SN too. In case, distance between NN and SN is in multi-path environment then NN looks for another NN to multi-hop its packets towards the SN. The reason behind avoiding long distance route is that chances of fading and attenuation of data signal are very high, hence the probability of packet loss is maximum. In order to minimize the aforementioned factors, shorter path is selected to relay the data packet towards the destination. The distance is checked via step 4 that ensures that data packets are transmitted over the path have receiver with in the communication range. After the computation of distance and finding forwarder node in transmission range, one of the most crucial factors of our proposed work is data traffic estimation at each hop to avoid overburden of data on the intermediate super nodes. The traffic load is estimated via using Equation (9) and average load is computed with the help of Equation (10). Also energy dissipation is considered along with the data traffic in order to pick optimal routing path that has less load and minimal energy consumption. Using Equations (7) and (8) both receiving and transmitting energies are computed before delivering data to next SN. At step 7, if the traffic is very high then SN is selected in near vicinity of NN for compensating high energy consumption with its heterogeneity nature. Then data traffic is transmitted to the SN until it reaches the sink. Step 15, at the completion of each round, overall energy consumption is computed in order to measure the network lifetime. All steps are repeated till the time no node in the network remains alive or is unable to deliver data at the destination due to the unavailability of intermediate nodes.

### 3.6. Energy Minimization

To portray reduction in the energy consumption once the data traffic load is estimated and distributed accordingly. We have carried out an optimization approach through which a feasible solution is computed. If the energy consumption satisfies the constraints then it is justified that data traffic is balanced. To prove it mathematically, linear programming is adopted. Linear programming is a mathematical approach which is broadly taken in to consideration while optimizing desired objective(s). The optimization process could be maximizing or minimizing depending upon the nature of objective function such as energy; it always needs to be minimized. An objective function must operate within the given linear constraints. We define the following objective function for the achieving optimal result in term of energy consumption.

(12) $$minimize\sum_{r=1}^{r_{max}}E_{tax}\left(r\right)\;\forall r\in r_{max}$$

Energy dissipation mainly includes the reception and transmission energies which are collectively known as energy tax $\left(E_{tax}\right)$. The energy tax increases as the number of hops or number nodes involve in data packet delivering increases. If we want to save the node battery power, one of the factors must need to be analyzed. Here, we have minimized the number of nodes by exploiting divided and conquer mechanism. Due to the division of the network field; nodes are restricted only to the specific sector. Now, Equations (7) and (8) are combined to find out the total energy dissipated in one round. Equation (13) is formulated to portray the overall amount of energy dissipated in delivering data packets successfully at the destination.

(13) $$E_{tax}\left(r\right)=E_{tx}+\sum_{n=0}^{N}\left(E_{tx}+E_{rcv}\right)\times \left(n\times P_{kt_{sink}}\right)\;\forall n\in N$$

$E_{tx}$ is separately added because nodes in sink’s range will forward data directly. On the other hand, second expression with summation is included to show both energies from upstream nodes toward the downstream nodes in the network field. *n* is zero because it is possible that due to death of nodes, few sectors could be empty. Now, to restrict our objective function, following constraints are defined.

**Constraints:**

(14) $$Con_{1}:\;\;E_{tx},E_{rcv}\leq \varepsilon_{o}\;\;\forall n\in N$$

(15) $$Con_{2}:\;\;E_{F}\leq E_{F}^{min}\;\;\forall n\in N$$

(16) $$Con_{5}:\;\;TR_{n}\leq TR_{n}^{max}\;\;\forall n\in N$$

The purpose of Equation (14) is to ensure that energy required for transmission and reception should be less than the initial energy $\varepsilon_{o}$ of the node. Similarly, $Con_{2}$ shows the constraint regarding the selection of forwarder data, that a node having less energy consumption as compared to near by nodes will be picked as a forwarder in order to optimize the network lifetime. For receiving good quality signal, it should be transmitted within the communication range which is exactly forced by Equation (16).

**Figure 3:**
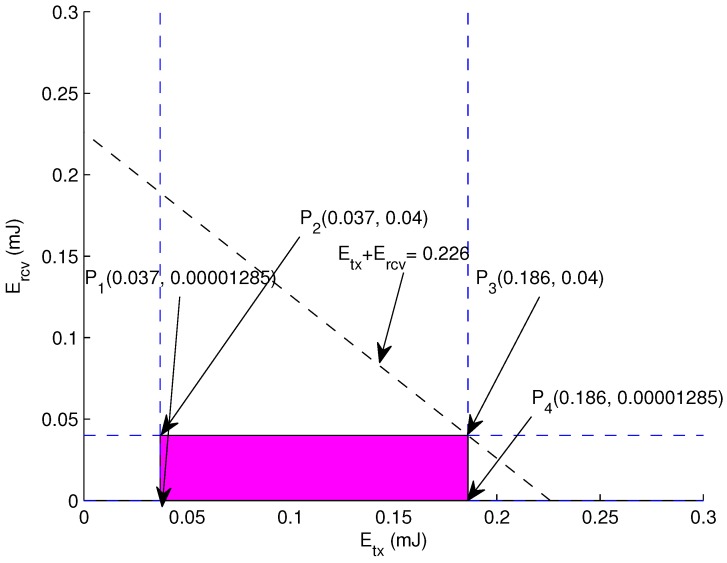
Feasible Region: Energy.

**Graphical Analysis:** The graphical analysis is presented because of its nature to provide clear visualization about precise indications of all possibilities within the bounded region. For two dimension scenarios, graphical approach is best fit to demonstrate optimal available options. The graphical analysis of our energy minimization is depicted in Figure 3.

Let assume $\varepsilon_{fs}$ = 20 nJ, $\varepsilon_{amp}$ = 20 nJ, $E_{elec}$ = 50 nJ, *d* = 60 m, *r* = 120 m, ρ = 10, ξ = 10, *x* = 5 m, θ = 45${}^{\circ }$, *R* = 500 m, *N* = 150. Following values are extracted via using aforementioned equations.

(17) $$0.037\leq E_{tx}\leq 0.186$$

(18) $$0.00001285\leq E_{rcv}\leq 0.04$$

(19) $$0.037\leq E_{tx}+E_{rcv}\leq 0.226$$

The enclosed region is calculated by keeping in mind the constraints given in Equations (14)–(16). Equations (17)–(19) are drawn to find out feasible region. An optimal solution is validated by each vertex of the feasible region. The solution can be validated from the points on the boundary of the feasible region. The points are:

$P_{1}$ (0.037, 0.00001285) = 0.037 mJ
$P_{2}$ (0.037, 0.04) = 0.077 mJ
$P_{3}$ (0.186, 0.00001285) = 0.186 mJ
$P_{4}$ (0.186, 0.04) = 0.226 mJ

Hence, it is validated that the traffic load is well distributed due to the deployment of heterogeneous nodes. The energy consumption within the bounded region will be optimal and contributes in enhancing the network lifetime.

### 3.7. Throughput Maximization

In this section, we maximize the network performance through efficient bandwidth allocation to super nodes. During the process, normal nodes send data to their respective super nodes in their transmission range. For the selection of SN as a data forwarder maximum bandwidth is required such that to forward data maximum amount of data. For the selection of SN, it is necessary that SN has high bandwidth in order to make sure that congestion is avoided and data is delivered to the sink successfully. Based on this, an objective function under linear constraints has been defined as follows:

(20) $$maximize\sum_{r=1}^{r_{max}}Th\left(r\right)\;\;\forall r\in r_{max}$$

where, Th(r) is computed like given in Equation (21)

(21) $$Th\left(r\right)=\sum_{r=1}^{r_{max}}Pkt\left(r\right)\;\;\forall r\in r_{max}$$

**Constraints:**

(22) $$Con_{1}:\;\;E_{n}\leq \varepsilon_{o}\;\;\forall n\in N$$

(23) $$Con_{2}:\;\;E_{F}\leq E_{F}^{min}\;\;\forall n\in N$$

(24) $$Con_{3}:\;\;TR_{n}\leq TR_{n}^{max}\;\;\forall n\in N$$

(25) $$Con_{5}:\;\;Max\sum_{sn=1}^{sn_{max}}B_{SN}\;\;\forall sn\in SN$$

Objective function in Equation (20) is used to maximize the throughput of the network. Constraint $Con_{1}$ and $Con_{2}$ illustrate that the energy consumption of nodes must be minimum. Moreover from Equation (23) it is clear that energy of forwarder node is less than the node with minimum energy in a specific region. Equation (24) states that transmission range of sensor nodes is within the maximum transmission range of sensor nodes. Equation (25) depicts the allocation of maximum bandwidth to SN and remaining is divided equally between NN.

Let *B* denotes the total bandwidth allocated to the network i.e.,:

(26) $$B=B_{NN}+B_{SN}$$

where $B_{NN}$ is the *B* allocated to NN and $B_{SN}$ is the *B* allocated to SN. The network area is divided into *N* coronas’ and further divided into *S* sectors. So the allocation of bandwidth in *S* can maximize the network performance. Let $B_{n}$ be the *B* allocated to a node in $C_{i}$ then:

(27) $$B_{NN}=B_{n}\times N_{nn}$$

Let assume in a network $N_{nn}$ ranges from $\left\{2,3,.....\frac{N_{nn}}{N}\right\}$ in *N* coronas network. For efficient routing, we must have at least two nodes in a sector and maximum of $\frac{N_{nn}}{N}$ nodes. Exceeding this value results in decreasing number of nodes in other sectors.

Also we have

(28) $$B_{n}=\frac{ATF}{t}$$

where ATF denotes average traffic load on sensor nodes which can be calculated from Equation (11).

(29) $$B_{SN}=B-B_{NN}$$

**Graphical Analysis:** Let assume total $N_{nn}$ = 110 and *N* = 10, *B* = 500 kHz to 1 MHz, *R* = 500 m, *r* = 60 m, ξ = 3 m, *x* = 5 m, *t* = 10 ms. For $N_{nn}$ = 2, ATF = 466.12 bits, $B_{NN}$ = 93.2 kHz and $B_{SN}$ = 406.8 kHz. For $N_{nn}$ = 11, $B_{NN}$ = 512 kHz and $B_{SN}$ = 488 kHz. Now Equations (26), (28) and (29) can be represented as:

(30) $$93.2\leq B_{NN}\leq 512$$

(31) $$406.8\leq B_{SN}\leq 488$$

(32) $$500\leq B_{NN}+B_{SN}\leq 1000$$

**Figure 4:**
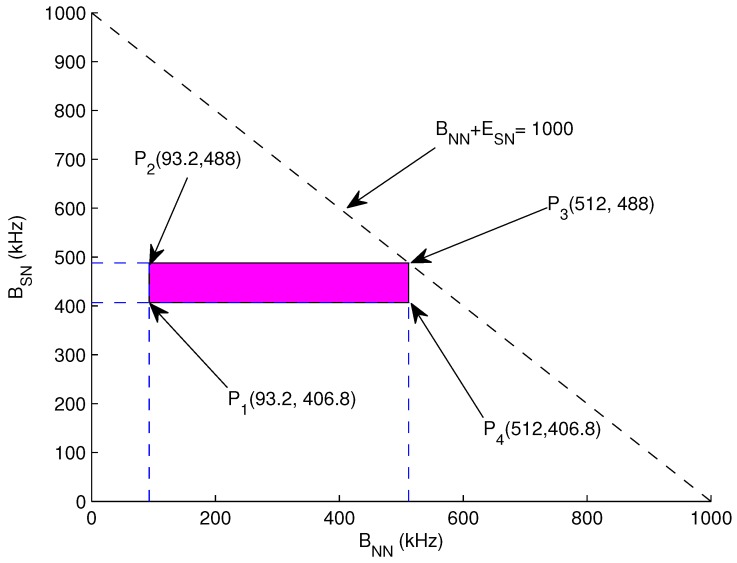
Feasible Region: Bandwidth.

The bounded region from the aforementioned equations is drawn in Figure 4 which is the feasible region of this bounded region.

$P_{1}$ (93.2, 406.8) = 500 kHz
$P_{2}$ (93.2, 488) = 581.2 kHz
$P_{3}$ (512, 406.8) = 918.8 kHz
$P_{4}$ (512, 488) = 1 MHz

These points validate a valid solution for lifetime maximization. Hence selecting any value from the bounded region for *B* allocation always results in increasing the lifetime of forwarder nodes.

### 3.8. Data Transmission

Once the traffic load is estimated in each sector and feasible solution for energy consumption is computed and rest of the pre-requisites are fulfilled then data transmission is carried out towards the respective destination. In converge-cast routing algorithm, nodes deployed in the upper layer always pass on data to the downstream node. Nodes in outermost sector transmit data to the SN in their communication range. Then SN finds out the SN from $C_{n-1}$ [12]. Due to the increment of load from top to bottom, heterogeneous network is considered. Let assume $NN_{j}$ sends data from sector $S_{n}$ to sector $S_{n-1}$ in order to transmit data to the sink as shown in Figure 2. Distance between them is $d_{\left(i,j\right)}$. If the distance is within the free space then data transmission proceeds else an alternative route is computed to deliver data at the destination or SN. Lets assume *k* bit data to be transmitted by the nodes in sector $S_{n}$ to the nodes in sector $S_{n-1}$. Energy consumes in sending *k* data to the next hop can be calculated from Equation (7).

The data transmitted by node *i* in sector $S_{n}$ to node *j* in sector $S_{n-1}$ is equal to $k\times ATF_{iM_{s}}$ and the data receive by node in sector $S_{n-1}$ is $ATF_{iS_{n}}-1$; NN in a sector $N_{n}$ interacts with normal node in sector $S_{n-1}$. If NN *i* in a sector is unable to find normal node *j* in sector $S_{n-1}$, void hole occurs. In that case normal node has to find a suitable forwarder node. SN in a sector receives the data from NNs in case when void hole occurs. The situation arises when normal node does not lie in transmission range of SN. Then NN sends the data to super node through its neighbor node which is close to SN. If somehow neighbor node in respective sector is not available, super node collects the data by itself from normal node. After the data successful reception by SN in a sector $S_{n}$, it transmits the data to SN in sector $S_{n-1}$. As the time progresses, after the death of super node in sector $S_{n}$, super node present in adjacent sector *S* acts as data forwarder for remaining nodes. It has enough energy for sending data to the sink directly and this process continues until all the data is successfully delivered to the sink.

## 4. Performance Evaluation

In this section, we evaluate the performance of our propose strategy through simulations in a simulator. LiMHA is compared with existing schemes ODTS, LAEHA, N-SEP, and P-SEP through simulations because all techniques have corona based models. Simulation parameters are listed in Table 2.

### 4.1. Performance Metrics

In this section, we use performance metrics for estimating the performance of the routing techniques.

### 4.2. Metrics Definition

**Network Lifetime (s):**

Network lifetime ℵ(t) is defined as the lifetime achieved by corona with maximal PNAEC in *t*-th iteration. i.e.,

(33) $$ℵ\left(t\right)=\frac{\varepsilon_{o}}{max\{E_{i}\left(t\right),i=1,2,...N_{c}\}}$$

where $\varepsilon_{o}$ is the initial energy of each node and $E_{i}\left(t\right)$ is the PNAEC of corona $C_{i}$ in the t-th iteration.

**Energy Tax (J):**

Energy tax is defined as the average energy consumption of a node during successful transmission of packets to the sink. Energy tax can be computed as follows:

(34) $$Energytax=\frac{E_{total}}{N\times Packets_{Sink}}$$

where $E_{total}$ denotes the total energy consumption of the network. N denotes total number of nodes in the network and $Packets_{Sink}$ denotes the number of packets receive by the sink.

**End to End delay (s):**

E2E is defined as the average time elapsed from the start when packet leaves source node until the packet successfully received by the sink.

### 4.3. Network Lifetime Comparison

**Figure 5:**
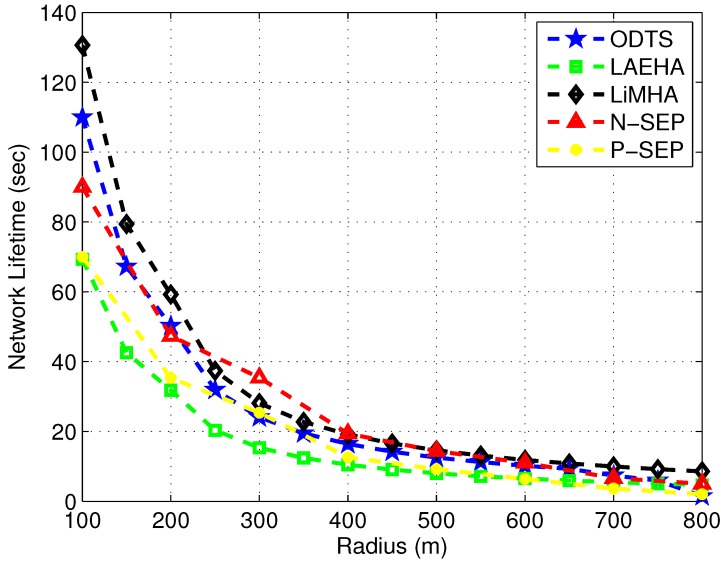
Network Lifetime at Various Radii.

Figure 5 compares the network lifetime under different radii. It is clear from figure that network lifetime decreases with the increase in network radius. This is due to increase in distance between nodes which increases the energy consumption. Moreover probability of void hole increases with increase in network radius which leads to short lifespan. LiMHA has 15% higher network lifetime as compared to ODTS on average and 45% improved lifetime as compared to LAEHA. In LiMHA network is divided into sectors and presence of each super node in a sector decreases the void hole probability. Super node has the capability to receive the data from normal nodes as well as to send the collected data from normal node to downstream super node. In the start when network radius ranges from 100–200 m, the performance of ODTS is observed better in terms of maximizing network lifetime except with the case of 100 m radius. ODTS uses ant colony optimization for achieving better lifespan in sparse network. It chooses a path that is more energy efficient as a result data is transmitted through the shorter path. For large area network the performance of ODTS in terms of lifetime degrades as compared to LiMHA. LiMHA uses super nodes for data transmission. If normal nodes unable to locate neighboring nodes in their transmission range they transmit the data to their respective super node in their sector without any hesitation. Thus probability of transmission of data increases which increases the overall network lifetime. With the increase in network area traffic load increases. In LiMHA, super nodes have enough energy to bear the burden of high traffic load, however in ODTS high traffic load on nodes decreases the network lifetime. Same is the case for LAEHA where high data load on nodes near the sink decreases the lifetime of sensor nodes due to high energy required for sending data to the sink. Thus network lifetime tends to decrease rapidly.

For better visualization, we have compared our protocol with cluster based heterogeneous schemes N-SEP and P-SEP. The network lifetime is the metric to validate the legitimacy and effectiveness or our proposed work with respect to the baseline strategies for energy efficiency. We have increased the transmission radius for all schemes for fair comparison, where it is evident that with the increase in radii, the network nodes have to transmit data over long distances, that is well known with the increase in distance directly proportional to energy tax. Authors in both N-SEP and P-SEP have used small communication distances, however, when we increased the communication distance for all schemes as shown in Figure 5. The network lifetime gradually starts declining due to uneven battery dissipation over long transmission ranges. Hence, it is well grounded that our proposed is suitable for both small and large communication ranges. While, other schemes perform well enough, but not as much needed.

### 4.4. Energy Tax Comparison

**Figure 6:**
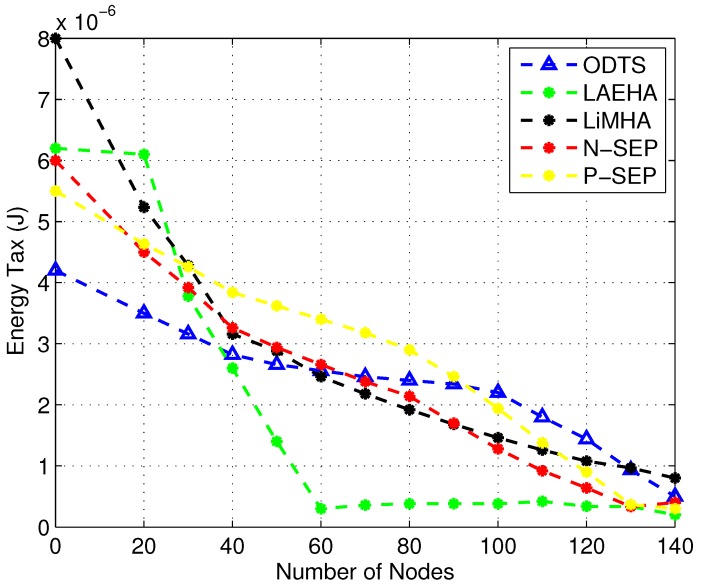
Energy Tax with Different Node ρ.

Figure 6 shows energy tax increases with the increase in node density. With number of nodes increases, distance between nodes decreases thus less energy is required for sending data to the sink. In sparse area network ODTS performs better as compared to others. ODTS chooses the most energy efficient and balanced path for sending data to the sink. Hence less energy is consumed. An average 26.5% less energy is utilized in ODTS is noted as compared to LiMHA. LAEHA has 41.3% higher energy tax as compared to ODTS in sparse area network. Due to less number of nodes in the network distance between nodes increases thus nodes have to send their data with high energy consumption which increases the overall energy consumption of nodes thus increases the energy tax as well. This distance decreases when there are enough number of forwarder nodes in the network due to which energy consumption for data transmitting and receiving is reduced. In case of dense network, overall energy consumption of the network is increased in ODTS due increase in neighbor requests with the increase in node density. Moreover in ODTS with the increase in number of nodes packet collision on ants effects the performance of network thus increases the energy consumption of the network. ODTS uses energy balanced distance for transmission of data hence overall energy consumption slightly decreases. On average LAEHA has less energy tax among the others i.e., averages 60.8% less energy tax as compared to LiMHA and 89.8% less energy tax when compares with LiMHA. LAEHA is energy efficient routing technique because it only considers forwarding of data with minimum distance to the sink.

For network having large number of nodes, LiMHA performance in terms of energy decreases as compared to LAEHA due to the fact that super node is selected as a data forwarder from a specific distance in order to overcome void hole. From the results of energy tax, it is understood that ODTS is more energy efficient in sparse network as compared to LiMHA but less energy efficient than both LiMHA and LAEHA in dense area network because of its ability of energy balancing which neither drop energy nor maximizes much.

Similarly, N-SEP has less energy tax as compared to ODTS and P-SEP when node density is low due to no redundant packets transmissions at the cluster head. However, as it can be seen that with the increase in node number, energy tax is decreased, however, in league with LiMHA, energy tax is high when nodes density is high. High energy dissipation is due to the overburden of traffic over the selected cluster head, while in LiMHA at each hop traffic load is estimated then data is transmitted. Due to ignorance of uneven traffic load, cluster heads deplete energy very quickly and lead to void hole. Clustering mechanism exploited in P-SEP is to maximize the network lifespan. Its energy consumption is higher than LiMHA , LAEHA, and N-SEP due to the random selection of cluster head at each round and the nomination of member nodes based on the minimum distance from the cluster head. Due to the optimal number of nodes association, few nodes having larger distance than the defined threshold unable to find cluster head. Resulting in wastage of energy leading to degrade the network performance. The energy dissipation also increased in P-SEP as node density increases in the network. The reason behind high energy tax is that due to the inefficient selection of cluster heads. There is the cyclic selection of cluster heads which becomes inappropriate when node ρ increases resulting in high data traffic at the cluster head and also no mechanism for cluster head congestion avoidance. Due to these factors, P-SEP is less energy efficient than LiMHA.

### 4.5. End to End delay Comparison

**Figure 7:**
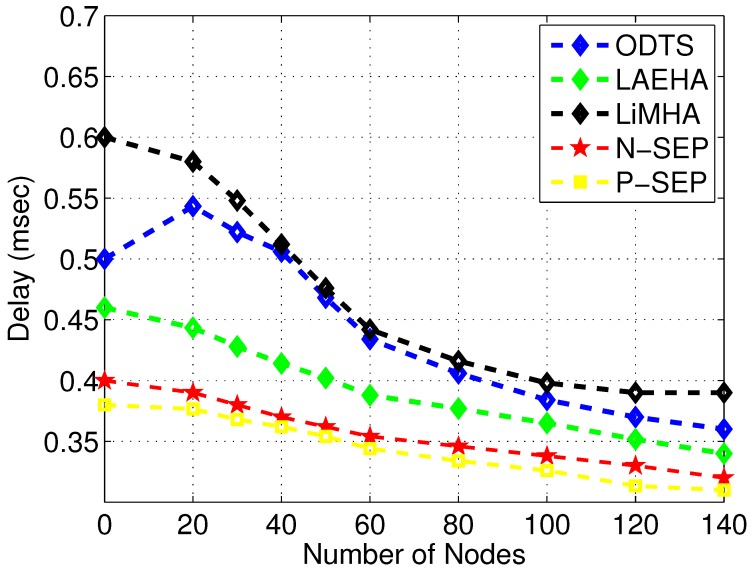
End 2 End delay.

Figure 7 leads to E2E delay comparison among LiMHA, ODTS, LAEHA, N-SEP and P-SEP. Contrary to energy tax, E2E delay decreases with increase in number of nodes. With number of nodes increases traffic load on lowest region increases thus sending high traffic load consumes lot of time which increases the E2E delay. Moreover packets collision between nodes increases the overall waiting time. ODTS has lower delay as compared to LiMHA. The main reason behind this with less number of nodes the distance between nodes and ants in a region increases thus ants have to wait for the packet to reach to it which increases the overall sending time but still better than LiMHA. Also due to less number of nodes probability of node failure tends to increase rapidly thus nodes have to choose other routing path for packet transmission. In term of achieving better results with minimum E2E delay in sparse area network LAEHA performance is better due to the following reason: LAEHA does not perform number of calculations for finding the forwarder nodes. However in LiMHA normal nodes in a sector have to decide whether to send the data to normal nodes in lower region or to forward the received data to super nodes in respective region. Moreover number of neighbor requests in LAEHA are less as compared to LiMHA and ODTS that add extra E2E delay into the routing technique with high number of neighbor requests. When network size grows up, the performance of ODTS is remarkable as compared to LiMHA in terms of achieving minimum E2E delay level because data is forwarded to best routing path which is most energy efficient path and this level increases on average as compared to LAEHA which has high level of minimum delay among others on an average. In LiMHA, the network area is divided into sectors and in each sector normal node has to choose its data forwarder such that the packet successfully reaches to its destination. Similarly, the E2E delay of P-SEP from all baseline schemes including N-SEP is minimum due to the factor of its greedy approach of selecting cluster head at an optimal distance from sink and member nodes with in the threshold value of communication range. That is the main factor its performance degrades as communication radii increases, however, its E2E delay remains smaller than rest of the counterpart schemes. N-SEP performs well with respect to baseline schemes excluding P-SEP as shown in Figure 7.

### 4.6. Performance Trade Off

In this section, we discuss the performance of of our propose scheme LiMHA with the selected schemes ODTS and LAEHA. The proposed scheme performs better in terms of optimizing network performance. The higher lifetime achievd by LiMHA is due to division of network into number of sectors and high energy node is present in each which increases the probability of successful transmission of data to to sink. On the other hand there is no such phenomena of super node deployment is discussed. However ODTS has better network lifetime as compared to LAEHA because ODTS uses ant colony optimization for transmission of data to the sink while LAEHA only considers commonly used transmission strategy. Looking at the energy comparison, LiMHA achieve less energy when compared with ODTS in dense area network. ODTS uses the most energy balance distance for transmission of data to the sink. It energy slightly changes with node density. In LiMHA, presence of each super node in a sector minimizes the energy of normal nodes due to selection of forwarder node at minimum distance thus normal nodes have to choose low distance nodes in their transmission range. With the deployment of super node the minimum distance level is increased. The performance of LAEHA in terms of less energy consumption is outstanding because LAEHA only considers the selection of forwarder node on minimum depth. E2E delay level of LiMHA is worst due to number of calculations involved in selecting the forwarder nodes. Normal node in a sector calculates its distance with neighboring normal node and super node. Also it calculates its distance with the down sector normal normal thus delay is increased in this scenario. In terms of achieving minimum delay level LAEHA performance is better compared to ODTS because ODTS considers the time involves in selection of best possible path for data transmission increases the packet sending time thus delays the performance of the network while there is no such phenomena is adopted in LAEHA. N-SEP achieved energy efficiency in sparse deployment and while effective network lifetime in sparse deployment, however, E2E delay is minimized due to the k-mean clustering algorithm. Similarly, P-SEP is attains minimum E2E delay and energy efficiency in sparse deployment due to the selection of cluster heads at optimal distance from sink and members having minimum distance from the respective head node. The performance trade off table is shown in Table 3.

## 5. Conclusions and Future Work

In this paper, we have proposed a transmission strategy that is able to alleviate hole in order to maximize the network lifetime. The deployment of heterogeneous nodes in each region proved to be successful in term of balanced data transmissions over an intermediate node. In addition, the division of the network field contributed enough in balancing the energy among the network nodes. Due to the election of the optimal forwarder at each hop, even energy dissipation is achieved. The existing technique ODTS is based on ACO which computes two functions for finding the optimal energy efficient route and minimal distance between source and destination. However, the estimation of data load at each hop in proposed algorithm proved to be very effective in selecting route to the destination. Through linear optimization, we have computed a feasible region which shows that energy consumption within that region always helps the network nodes to operate for the maximal time. Moreover, after the death of an intermediate node, transmission range is adjusted in order to transmit data packets directly to the destination. This also increased the network lifetime and allows the node to utilize its energy resources completely. The effectiveness and validity of the proposed work are shown through simulations. The results are portrayed in which it is evident that proposed work outperformed selected existing counterpart scheme in terms of network lifetime and energy consumption.

In future, we will extend our work in different ways. Firstly we will do random deployment of heterogeneous nodes in the network, i.e., they can be placed anywhere in a sector. Secondly, we will find out an optimal number of nodes that are capable of delivering data to intermediate nodes. 

## Figures and Tables

**Table 1:** Comparison of the State of the Art in WSNs.

Protocol	Technique Used	Metrics	Parameters Achieved	Parameters Compromised
HORA [14]	Multi-hoping	Energy and distance	Network lifetime increases	Transmission Delay increases, high energy consumption
LBT [15]	Multi hop ring routing	Energy and Distance	Network lifetime maximizes	Energy Hole
NEHA [16]	Sleep schedule mode, multi-hoping	Energy and Distance	Network lifetime is maximized	High traffic load on boundary nodes increases energy consumption, E2E delay is increased
E-HORM [18]	Sleep schedule mode is adopted, multi-hoping	Energy threshold, distance	Network lifetime maximizes	E2E delay increases
IHDHRA [19]	Node replacement	Forwarder function and energy	Network lifetime is increased	Scalability and Robustness
EEDSRS [21]	Data sensing and Data routing, rate allocation	Link Quality	Maximum network lifetime is achieved	MAC layer protocol
EEAC [24]	Ant colony optimization	Energy	Network lifetime increases with less energy consumption	Scalability and Robustness
ODTS [25]	Ant colony optimization	Distance and Energy	Network lifetime is optimized	Performance degraded in dense area network
EEDCP-TB [30]	Data gathering Tree based Routing	TDMA	Network lifetime optimization through energy minimization	E2E delay increases
LMDE [27]	Multi hop Routing	Energy	Network performance optimizes	Scalability issue and Data Redundancy

**Table 2:** Control Parameters.

Parameters	Value
Number of Coronas	10
Total Number of Nodes	150
Number of Normal Nodes	150
Number of Super Nodes	40
Number of Sectors	40
Initial Energy of Normal Nodes (J)	1
Initial Energy of Super Nodes (J)	1.5–6
Transmission range (m)	60
Radius (m)	100–800
Number of rounds	10
v (m/s)	3 × $10^{8}$

**Table 3:** Performance Trade Off.

Protocol	Technique	Metrics	Achievement	Cost to Pay
LiMHA	Mixed Routing	Distance and Energy	Network lifetime prolongs	High E2E delay
ODTS	Mixed Routing	Distance and Energy	Higher network lifetime and less energy for sparse case,	High E2E Delay for sparse case
LAEHA	Mixed Routing	Distance and Energy	Less Energy Consumption in dense case, Minimum E2E delay level is increased	Less network lifetime
N-SEP	Cluster Routing	Distance and Energy	High Energy Dissipation in High Communication Radii	Less network lifetime
P-SEP	Cluster Routing	Distance and Energy	Minimum E2E Delay	Less network lifetime
